# Death with graft function after kidney transplantation: a single-center experience

**DOI:** 10.1007/s10157-017-1503-9

**Published:** 2017-11-20

**Authors:** Mi-yeon Yu, Yong Chul Kim, Jung Pyo Lee, Hajeong Lee, Yon Su Kim

**Affiliations:** 1 Department of Internal Medicine, Seoul National University Hospital, Seoul, Korea; 2 Department of Internal Medicine, Seoul National University Boramae Medical Center, Seoul, Korea; 3 Kidney Research Institute, Seoul National University, Seoul, Korea; 4 Department of Medical Science, Seoul National University College of Medicine, 101 Daehak-ro, Jongno-gu, Seoul, 03080 Korea

**Keywords:** Death with graft function, Kidney transplant, Infection, Malignancy

## Abstract

**Background:**

Death with graft function (DWGF) is an important cause of long-term loss of grafts and patients. In this study, we investigated clinical characteristics and causes of DWGF in kidney transplant recipients.

**Methods:**

We recruited kidney allograft recipients who underwent surgery during 1973–2016 at Seoul National University Hospital in Korea (*n* = 2137). We divided recipients into four groups: alive with graft function (AWGF), alive with graft loss (AWGL), DWGF, and death with graft loss (DWGL).

**Results:**

Among 455 recipients with graft loss, 88 (19.3%) lost graft function due to death. DWGF was responsible for 38.6% of a total of 228 deaths. Recipients with DWGF were older, more often diabetic, and experienced delayed graft function more often compared to patients with AWGF, AWGL, and DWGL. Additionally, they had fewer episodes of acute rejection than AWGF and AWGL patients. The majority of DWGF developed because of infection (40.9%), malignancy (28.4%), and cardiovascular disease (11.4%). Infection-related mortality was highest within the first year after transplantation. Death due to malignancy was lowest within the first year, but increased thereafter.

**Conclusions:**

In our center, DWGF was a significant cause of graft loss. Infection and malignancy were the leading causes of DWGF during the overall post-transplantation period. Therefore, close monitoring for infection and malignancy should be instituted to lessen the burden of graft loss.

## Introduction

Death with graft function (DWGF) is the leading cause of long-term graft failure [1]. Despite improvement in short-term transplantation outcomes, long-term transplantation outcomes need to be improved, especially regarding graft function. DWGF develops consistently during the first 5 years after transplantation and increases 5–10 years after transplantation, even though graft failure has decreased steadily over time [2, 3]. Previous studies demonstrated that DWGF accounted for 42% of all graft failures within the first year after kidney transplantation (KT), and 54% within the first 10 years after transplantation [3]. Therefore, reducing DWGF is crucial for improving KT outcomes.

In previous studies, the main cause of DWGF was cardiovascular disease (CVD) [4–8]. Uncontrolled blood pressure, hypoalbuminemia, anemia, delayed graft function (DGF), HLA mismatches, and higher steroid doses were associated with DWGF incidence [4–7]. Higher blood pressure, hypoalbuminemia, and anemia are well-known risk factors for CVD. In addition, a higher degree of immunosuppression is related to not only DGF development, but also increased future CVD risk after transplantation [9–11]. Although CVD incidence and outcome are different according to race [12], data regarding Korean allograft recipients are lacking. Therefore, it was necessary to determine the accurate causes of DWGF in an Asian population.

DWGF in kidney recipients is common. Therefore, it is important to identify the causes of and risk factors for DWGF, because increasingly more KTs. The time period after KT may affect the cause of death in recipients, because the degree of immunosuppression and its cumulative effect may be different based on time. Therefore, studies are needed to identify the causes of DWGF according to the period after transplantation.

Understanding the main cause of DWGF and the changes in possible causes may help control DWGF; the final goal is improving long-term graft survival. In this study, we investigated the causes of DWGF and evaluated those causes depending on the time period after transplantation.

## Materials and methods

### Patients

Our retrospective study cohort consisted of adult and pediatric patients who received a kidney allograft during 1973–2016 at Seoul National University Hospital in Korea. We excluded recipients without precise information regarding graft function or recipient survival. We divided the patients into four groups according to graft function and recipient survival: alive with graft loss (AWGL), alive with graft function (AWGF), DWGF, and death with graft loss (DWGL).

### Clinical characteristics

Clinical information was extracted from electronic databases at our center. These data included both recipient and donor characteristics. Demographic factors such as age and sex, causes of end-stage renal disease, dialysis duration and modality, transplant number, transplantation era, and multi-organ transplants were gathered from recipients. In addition, donor characteristics, such as age, sex, underlying diabetes at the time of transplantation, and donor types, were obtained. Data regarding transplant-related characteristics, such as ABO mismatches, HLA mismatches, DGF development, and biopsy-proven acute rejection (BPAR) episodes, were also collected.

### Graft loss and mortality

Graft losses were investigated from medical records and the national renal replacement therapy database maintained by the Korean Society of Nephrology [13]. Data regarding mortality, death date, and causes of death were obtained through a review of medical records and death certificates. In addition, we obtained mortality data of patients who were lost to follow-up from *Statistics Korea* using unique identifiers that were extracted until December 2014.

### Definitions and measurements

DWGF was defined as death of a kidney allograft recipient who had preserved kidney function without the need for dialysis or re-transplantation. We excluded acute kidney injury (AKI)-related dialysis events from DWGF [14]. Baseline creatinine was defined as the lowest value within 6 months before the last visit. We divided causes of death into six categories: CVD/stroke, infection, malignancy, chronic renal failure, other, and unknown. Changes in causes of death were assessed within 1 year after transplantation, from 1 to 5 years after transplantation, from 5 to 10 years after transplantation, and more than 10 years after transplantation. Transplantation era was divided into three categories based on the date of transplantation (era 1: 1973–1995; era 2: 1996–2005; and era 3: 2006–2016). Multi-organ transplantation was defined as transplantation of two or more organs, including both simultaneous transplantation and transplantation with intervals. DGF was defined as requiring dialysis during the first week after transplantation. BPAR was determined according to the 2007 Banff classification with biopsy performed before 2014 [15], and according to the 2013 Banff classification with biopsy performed after 2014 [16].

### Statistical analysis

All analyses were performed with SPSS 21.0 (SPSS Inc., Chicago, IL). Parametric variables were provided as means and standard deviation (± SD). Non-parametric variables were provided as median and interquartile range. Continuous data were compared using the Student’s *t* test or Mann–Whitney test. Categorical variables were compared according to their proportions found using the Chi-square test.

Cox logistic regression was used to identify risk factors related to DWGF compared with AWGF. Proportional hazards assumptions for Cox models were tested using log-minus-log plots. We chose several covariates found to be statistically significant by a univariate Cox hazard ratio analysis and retained them as potential predictors. A stepwise multivariate Cox regression analysis was performed to assess hazard ratios for DWGF after adjustment for recipient age, sex, pre-transplantation diabetes, dialysis duration, history of KT and multi-organ transplantation, donor age and type, and the number of HLA mismatches. To determine risk factors for DWGF compared with DWGL, we used logistic regression analysis.

## Results

### Study subjects

**Fig. 1:**
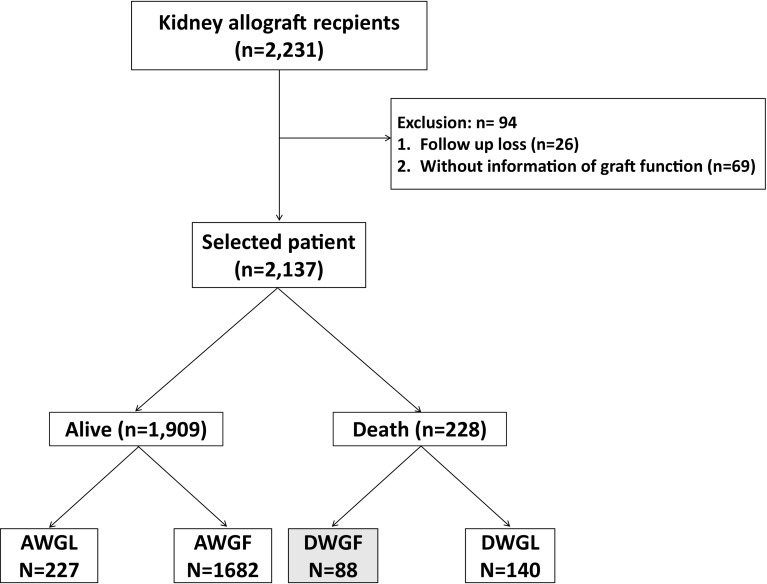
Algorithm for eligible patient selection. *AWGL* alive with graft loss, *AWGF* alive with graft function, *DWGF* death with graft function, *DWGL* death with graft loss

A total of 2137 patients received kidney transplants and were enrolled. Among them, 228 (10.2%) patients died and 455 (20.3%) patients had graft loss. DWGF occurred in 88 patients, accounting for 38.6% of patients who died and 19.3% of patients with graft loss. AWGF, AWGL, and DWGL patients comprised 78.7, 10.7 and 6.6% of total allograft recipients, respectively (Fig. 1).

### Comparisons of clinical characteristics between the groups

Median age of DWGF patients was 47.5 (36.0–57.8) years. More than 70% were men, who received their allograft from living donors. Pre-transplant diabetes cases were found in 23.9% of DWGF patients. Eighty-three percent of DWGF patients received hemodialysis before KT. Median dialysis duration was less than 1 year. Median time to death was 66.1 (11.3–148.9) months.

Next, we compared the clinical characteristics of DWGF patients with other groups (Table 1). Compared to the other three patients, DWGF patients were the oldest, had the highest number of patients with diabetes, and the highest number of patients with of DGF. Compared to AWGF patients, DWGF patients had shorter dialysis durations and lower incidences of BPAR. Compared to AWGL patients, DWGF patients received more allografts more often from deceased donors and had previously experienced a kidney transplant. DWGF patients had more HLA mismatches, but BPAR were fewer than the AWGL group. Compared to DWGL patients, DWGF patients experienced longer dialysis durations, and more often received allografts from deceased donors. There was no difference in the incidence of acute rejection between the two groups.

**Table 1:** Characteristics of the study population

Characteristics	DWGF	AWGF		AWGL		DWGL	
	(*n* = 88)	(*n* = 1682)	*P*	(*n* = 227)	*P*	(*n* = 140)	*P*
Recipient factor							
Age, year	47.5 (36.0–57.8)	40 (27.0–51.0)	< 0.001	26.0 (16.0–34.0)	<0.001	32.0 (21.5–43.0)	<0.001
Sex, male, no. (%)	63 (71.6)	1043 (62.0)	0.067	153 (67.8)	0.519	98 (70.0)	0.797
Primary cause of ESRD, no. (%)							
Diabetes	21 (23.9)	243 (14.4)	0.025	19 (8.4)	<0.001	11 (7.9)	0.001
No diabetes	67 (76.1)	1439 (85.6)		208 (91.6)		129 (92.1)	
Transplant, no. (%)							
First transplant	81 (92.0)	1584 (93.2)	0.488	224 (98.7)	0.009	134 (95.7)	0.245
Subsequent transplant	7 (8.0)	98 (6.8)		19 (1.3)		6 (4.3)	
Time on dialysis, months	10 (2.0–41.5)	24 (5.0–64.0)	0.002	6.0 (2.0–18.7)	0.225	5 (1.0–15.0)	0.038
Pre-transplant dialysis modality, no. (%)							
Preemptive transplantation	7 (8.0)	245 (14.6)	0.096	12 (5.3)	0.372	14 (10.0)	0.603
Hemodialysis no./HD + PD no. (%)	67/80 (83.8)	1048/1682 (62.3)	0.068	176/215 (81.9)	0.864	110/126 (87.3)	0.479
Peritoneal dialysis no./HD + PD no. (%)	13/80 (16.2)	634/1682 (37.7)		39/215 (18.1)		16/126 (12.7)	
Multi-organ transplant, no. (%)	3 (3.4)	63 (3.7)	1.000	2 (0.9)	0.107	2 (1.4)	0.377
DGF	10 (11.4)	45 (2.7)	<0.001	10 (4.4)	0.023	5 (3.6)	0.021
Transplantation era, no. (%)			<0.001		<0.001		<0.001
1973–1995	37 (42.0)	141 (9.8)		135 (59.7)		113 (80.7)	
1996–2005	24 (27.3)	306 (21.3)		66 (29.2)		19 (13.6)	
2006–2016	27 (30.7)	989 (68.9)		25 (11.1)		8 (5.7)	
Time to death, months	66.1 (11.3–148.9)					106.6 (21.1–196.2)	0.059
Time to graft loss, months	64.3 (9.3–143.9)			83.0 (36.0–144.0)	0.192	43.7 (6.8–113.1)	0.046
Donor factor							
Age, year	38.5 (29.3–51.0)	42.0 (31.0–50.0)	0.404	40.0 (29.5–49.0)	0.744	39.0 (27.0–52.0)	0.706
The age difference between donor and recipient, year	5 (− 6, 17)	0 (− 14, 7)	<0.001	− 13 (− 27, 2)	<0.001	− 5 (− 24, 8)	<0.001
Sex, male, no. (%)	49 (55.7)	906 (53.9)	0.896	108 (47.6)	0.197	68 (48.6)	0.296
Underlying diseases, no. (%)							
DM	1 (1.2)	4 (0.2)	0.221	0 (0)	0.108	0 (0)	0.386
Hypertension	0 (0)	4 (0.2)	1.000	0 (0)	–	1 (0.7)	1.000
Donor type, no. (%)							
Cadaver donor	27 (30.7)	550 (32.7)	0.801	36 (15.9)	0.003	18 (12.9)	0.001
Living related donor no./total LD no. (%)	53/61 (86.9)	866/1,132 (76.5)	0.077	172 (90.1)	0.486	100 (82.0)	0.397
Living unrelated donor	8/61 (13.1)	266/1,132 (23.5)		19 (9.9)		22 (18.0)	
Transplant-related factor							
ABO mismatches, no. (%)	3 (3.4)	68 (4.0)	1.000	1 (0.4)	0.035	1 (0.7)	0.301
HLA-A + B+DR mismatches no./total no. (%)							
0–3	40/66 (60.6)	1,043/1628 (64.1)	0.566	126/163 (77.3)	0.010	54/68 (79.4)	0.017
4–6	26/66 (39.4)	585/1628 (35.9)		37/163 (22.7)		14/68 (20.6)	
BPAR no. /total no. (%)	30/85 (35.3)	773/1672 (46.2)	0.048	109/227 (48.0)	0.027	51/142 (35.9)	0.925

Values are expressed as median (interquartile range) for non-parametric variables. Comparisons were made using the Chi-square test for categorical variables and the Mann–Whitney *U* test for continuous variables

*AWGF* alive with graft function, *BPAR* biopsy-proven antibody medicated rejection, *DGF* delayed graft function, *DWGF* death with graft function, *DWGL* death with graft loss, *LD* living donor

### Cause of death with graft function and graft loss

Table 2 shows the causes of DWGF. Infection was the most common cause of death (40.9%). The second most common cause of death was malignancy (28.4%). CVD/stroke-related mortality was the third most common (11.4%), with seven cardiovascular deaths and three stroke deaths. This distribution was significantly different from that of DWGL recipients, who most commonly died from chronic renal failure. Other causes included five deaths from accidents, four deaths from hepatic failure, two deaths from postoperative complications, and one death from suicide. Four patients who died from hepatic failure were recipients of only kidney transplants.

**Table 2:** Causes of death among kidney transplant recipients

	DWGF (*N* = 88) *N* (%)	DWGL (*N* = 140) *N* (%)
Cardiovascular/stroke	10 (11.4)	26 (18.6)
Infection	36 (40.9)	25 (17.9)
Malignancy	25 (28.4)	18 (12.9)
Chronic renal failure	0 (0)	31 (22.1)
Other	12 (13.6)	25 (17.9)
Unknown	5 (5.7)	15 (10.7)

*DWGF* death with graft function, *DWGL* death with graft loss

**Fig. 2:**
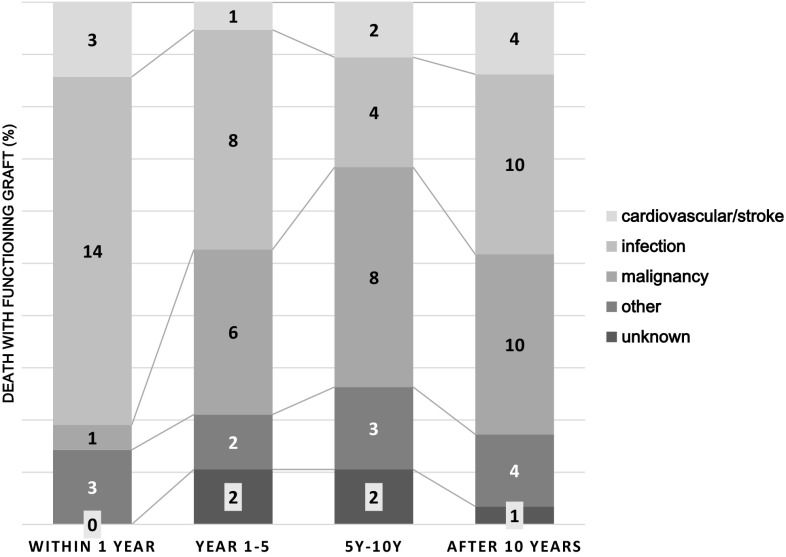
Cause of death with graft function within 1 year, 1–5 years, 5–10 years, and 10 years or more after kidney transplantation

Distributions of DWGF causes changed according to time after transplantation (Fig. 2). Infection-related mortality was highest at 66.7% within the first year after transplantation. Then, the proportion of them mortality decreased to 42.1% between post-transplantation years 1 and 5, and to 21.1% during post-transplantation years 5 through 10. Malignancy was the least common cause of death among the three main causes of DWGF within the first year. However, the proportion of cancer deaths increased gradually and became the most common cause at 5 years after transplantation. On the contrary, CVD/stroke mortality occurred constantly throughout the post-transplant period.

**Fig. 3:**
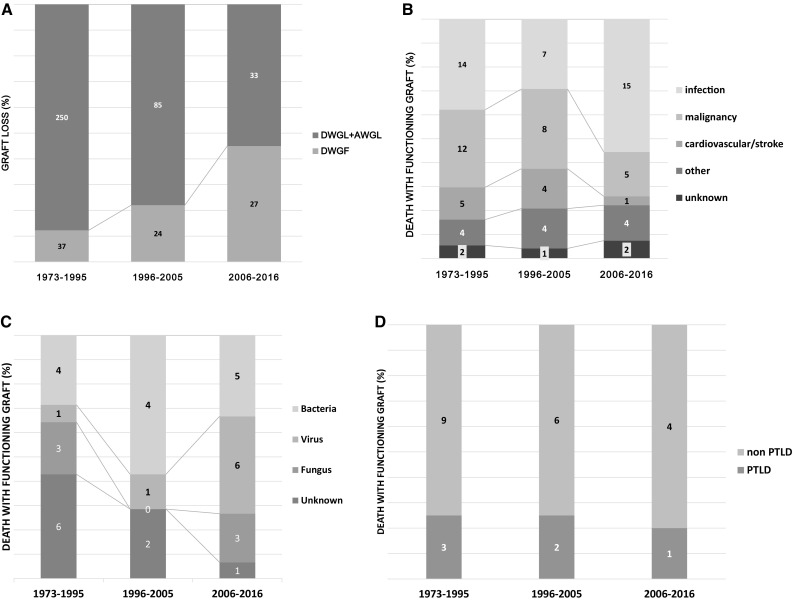
DWGF according to transplant area (**a**) proportion of DWGF in total graft loss (**b**) cause of DWGF (**c**) causative agents in infection-related DWGF (**d**) causative organ in malignancy-related DWGF. *DWGF* death with graft function, *PTLD* post-transplant lymphoproliferative disease

The incidence of graft loss decreased during the past 30 years. However, DWGF accounted for 12.9% of all graft failure during era 1; recently, this gradually increased to 45% during era 3 (Fig. 3a). Approximately 40% of DWGF patients who received a transplant during era 1 died due to infection (Fig. 3b). The proportion of infection-related mortality decreased slightly in era 2, but increased again during era 3. In particular, the proportion of viral infection increased gradually (Fig. 3c). In contrast, cancer-related and CVD/stroke-related mortality were significantly decreased recently. DWGF-associated post-transplant lymphoproliferative disease (PTLD) decreased over time (Fig. 3d).

We further investigated infection characteristics in DWGF (Table 3). More than half of infections attacked the lung. Soft tissue infection, including osteomyelitis, occurred in four patients. Brain infection was found in two pediatric patients. We could not find the cause of six (16.7%) infection-related deaths. Although lung infection was also most prevalent in DWGL patients, they did not die due to genitourinary or brain infections. Regarding causative organisms, bacterial infections were the most common, followed by viral and fungal infections.

**Table 3:** Characteristics of infection leading to death

	DWGF (*N* = 36) *N* (%)	DWGL (*N* = 25) *N* (%)
(A) Infected organ		
Respiratory tract	19 (52.8)	8 (32.0)
Genitourinary tract	2 (5.6)	0 (0)
Gastrointestinal tract	2 (5.6)	2 (8.0)
Brain	2 (5.6)	0 (0)
Other	5 (13.9)	2 (8.0)
Unknown	6 (16.7)	13 (52.0)
(B) Causative agent		
Bacteria	13 (36.1)	2 (8.0)
Virus	8 (22.2)	4 (16.0)
Fungi	6 (16.7)	3 (12.0)
Unknown	9 (25.0)	16 (64.0)

*DWGF* death with graft function, *DWGL* death with graft loss

Malignancy was the second most common cause of death and increased over time after transplantation. PTLD was the most common malignancy in DWGF patients. However, renal cell carcinoma was the main cancer for DWGL patients (Table 4).

**Table 4:** Origins of malignancy leading to death

	DWGF (*N* = 25) *N* (%)	DWGL (*N* = 18) *N* (%)
PTLD	6 (24.0)	4 (22.2)
Kidney	3 (12.0)	5 (27.8)
Liver	3 (12.0)	3 (16.7)
Stomach	3 (12.0)	2 (11.1)
Colon	2 (8.0)	1 (5.6)
Kaposi sarcoma	3 (12.0)	1 (5.6)
Other	5 (20.0)	2 (11.1)

*PTLD* post-transplant lymphoproliferative disease

### Risk factors for death with graft function

During the median follow-up (65.5 months), DWGF developed in recipients with older age [adjusted hazard ratio (HR), 1.064; 95% confidence interval (CI), 1.043–1.086; *P* < 0.001], pre-transplantation diabetes (HR, 2.0; CI, 1.136–3.519; *P* = 0.016), and DGF (HR, 3.757; CI, 1.913–7.377; *P* < 0.001) compared to AWGF patients. Compared to DWGL, DWGF was more prevalent in older recipients (adjusted OR, 1.058; CI, 1.037–1.080; *P* < 0.001).

## Discussion

In this study, we demonstrated that infection was the most common cause in DWGF patients, followed by malignancy. Unexpectedly, cardiovascular death comprised only 10% of overall causes of death in our cohort. Infection was most prevalent during the early post-transplantation period. Although infection-related death decreased slightly over time, it was the main cause of death, even 10 years after transplantation. Malignancy was the most cause during the late post-transplantation period. We also found that recipients who were older, and had pre-transplantation diabetes and DGF should be closely monitored for infection and malignancy events, even though they have preserved renal function.

DWGF developed in recipients with older age, pre-transplant DM, and DGF compared with the other three groups. These factors were independently associated with DWGF. Vulnerable immunity, ischemia reperfusion injury, and immunosuppressive therapy are regarded as risk factors for DWGF in recipients with older age, pre-transplant DM, and DGF [5, 17]. Furthermore, age was associated with DWGF using Cox multivariate analysis. Patients older than 65 years have undergone more KTs recently [2]. The median age of recipients was increased in the most recent era. However, older graft transplants have not increased as much as older recipients have. The gap between the shorter lifespan of older recipients after and the longer graft survival of younger grafts might result in higher DWGF risk. Lee et al. suggested that it is better to reduce the age difference between recipients and donors for good graft survival as well as reducing the risk of DWGF [18].

Overall, infection was the leading cause of DWGF in this study, whereas CVD is the most common cause of DWGF in western countries. The most common infection after KT is pneumonia [19, 20]. We also showed that the lung was the chief organ affected by post-transplantation infection in DWGF patients. Pneumonia in immunosuppressed patients is not easily detected at an early stage. Therefore, patients who visit the hospital with upper respiratory symptoms often have progressive pneumonia and high mortality. This may result in death without the loss of kidney function. Bacteria were the main causative agents of death. The most common organ targeted by bacterial infections was the lung (53.8%), but bacterial infections sometimes occurred in other organs such as the soft tissue, heart, or liver. Therefore, efforts to find an accurate primary infection are needed. Although bacterial infection is most common throughout the period, by era, viral infections gradually increased and were the most commonly observed in era 3. There are two main reasons for this. The first is that we did not know, in the past, what the cause of the infection was, but because of improvements in inspection technology, we can diagnosis the causative agents, especially viral infection. The second reason is this infection is associated with the use of immunosuppressive therapy [21, 22].

Interestingly, despite medical developments, the proportion of infection increased more in era 3 than in eras 1 and 2 among the causes leading to the death of DWGF patients. In our study, median age increased from 40 years in era 1 to 60 years in era 3. The drug effects in older recipients are different from that in younger [23]. Because of their pharmacokinetic and pharmacodynamic changes, the immunosuppression doses may be too high for older recipients. As age advances, the immune system is reconstituted and declines substantially, affecting survival [24, 25]. This immune senescence could put older recipients at a higher risk of infection. Additionally, the mortality risk of this infection is three times higher in elderly than young adult patients [26]. To avoid DWGF, there have been many reports about ways to reduce the infection risk, such as early immunosuppressant reduction, low steroid dosage, and the use of antimicrobial prophylactics [7, 20, 27]. We suggest early immunosuppressant tapering, especially steroid and continuous monitoring for infection, which may help to reduce DWGF by reducing infection risk. Pre-transplant immunization and post-transplant prophylactics should be considered for all potential transplant recipients. Furthermore, we need to recognize the diversity of drug responses, and the weakness of the immune system in the elderly.

We know that kidney transplant recipients are at higher risk for development of malignancy than the general population [28]. During the late period after transplantation, malignancy was the main cause of DWGF in this study. Malignancy was associated with intensive immunosuppressive therapy after KT and concomitant viral infection [29, 30]. The incidence of PTLD in renal transplant recipients is 1–5% [31]. Several factors, such as age, Epstein-Barr virus—negative recipient, and immunosuppressive therapy, were regarded as risk factors of PTLD [32–34]. PTLD was the most common cause of malignancy-related DWGF in this study; however, we found the proportion of PTLD had decreased slightly from 25% in era 1–20% in era 3. This decrease is the result of early detection of PTLD development and novel therapy [35]. However, we should provide continuous monitoring, early detection, and early treatment of PTLD, because the average age of recipients has increased and more elderly patients receive kidney transplants, despite new therapies and early detection of PTLD. The clinical practice guidelines committee of the American Society of Transplantation provides guidelines for cancer screening in kidney transplant recipients [36]. However, these guidelines were published in 2000, and there are no guidelines for KT in Asian populations. Therefore, new guidelines to screen for malignancy and to modify the risk factors before and after transplantation are needed. The importance and influence of these efforts have been growing over time.

In previous studies [4–8], cardiovascular deaths comprised approximately 24–30% of the total DWGF events, whereas CVD deaths comprised 11.4% in this study. Racial specificity, lower insurance costs, and routine examinations for CVD before and after transplantation may reduce the incidence of CVD-related DWGF in Korea. The Organization for Economic Cooperation and Development (OECD) announced that the mortality rate for CVD in Korea was 182 per 100,000 populations in 2011, which is lower than the OECD average [37]. Moreover, lower serum cholesterol and lower BMI, which could contribute to a lower risk of CVD, compared to that in western countries, may have contributed to this gap.

Our study has limitations inherent to its retrospective nature. Our study was a single-center study and involved patients who underwent transplantation over the course of several eras of different immunosuppression protocols spanning five decades. Despite these limitations, our study has strengths. First, we evaluated the causes of DWGF according to the time point after transplantation using a long observation period. Because the short-term outcome of transplantation has improved, graft survival is longer than ever. Therefore, there is more demand for evaluating time-dependent causes. Second, this study included high-risk kidney recipients, such as those with multiple transplants and second transplants, while the majority of other studies analyzed only low-risk patients.

In conclusion, infection and malignancy were the main causes of DWGF during the overall post-transplantation period, whereas CVD was the most common cause of DWGF in western countries. Infection was the most common cause of DWGF within the first post-transplantation year, whereas malignancy was the main cause of late DWGF. Our study suggests that efforts to give attention to causes, according to the time after transplantation, will improve the long-term outcomes and that new guidelines are needed for Asian populations.
